# FAMILY CAREGIVER PERSPECTIVES ON THE PALLIATIVE CARE NEEDS OF HOSPITALIZED PERSONS LIVING WITH DEMENTIA

**DOI:** 10.1093/geroni/igae098.0666

**Published:** 2024-12-31

**Authors:** Jung Kwak, Elizabeth Kvale, Sarah Mills, Kwaku Duah Oppong

**Affiliations:** The University of Texas Austin, Austin, Texas, United States; Baylor College of Medicine, Houston, Texas, United States; The University of Texas Austin, Austin, Texas, United States; The University of Texas Austin, Austin, Texas, United States

## Abstract

The progressive nature of dementia results in significant symptom burden, functional decline, and frequent hospitalizations, underscoring the need for specialized palliative care for persons living with dementia (PLWDs) and their caregivers. Existing studies, primarily retrospective analyses of health records or provider interviews, lack information on the critical psychosocial and social support contexts that significantly influence the palliative care needs of PLWDs. Addressing this gap, our study explored the perspectives of family caregivers on the palliative care needs of hospitalized PLWDs, informed by the Adapted Conceptual Model Integrating Palliative Care in Serious Illness and Multiple Chronic Conditions. We interviewed 37 caregivers of hospitalized PLWDs who were referred for palliative care. Caregivers identified primarily as female (66%) and caring for parents (58%), and as White (46%), Black (17%), Asian (5%), and Latino (31%). Most common challenges caregivers reported were about addressing health issues (58%), managing emotional difficulties (53%), and making care plan decisions (42%). Three related themes emerged from in-depth qualitative interviews: the complex care needs of PLWDs, including pain and symptom management; caregivers’ needs for support in discharge preparation; and challenges in making care transition decisions into facilities. These insights underscore the caregivers’ critical role in managing care and making decisions during critical care transitions for hospitalized PLWDs. The findings provide implications for practice and research including enhanced care coordination between primary, palliative, and discharge planning services in acute settings and future research on the impact of psychosocial and social support factors on palliative care outcomes for this vulnerable population.

